# Clot composition analysis in ischemic stroke of cancer patients: a systematic review

**DOI:** 10.1186/s12883-026-04739-2

**Published:** 2026-02-20

**Authors:** Athina-Maria Aloizou, David-Dimitrios Chlorogiannis, Kalliopi Pitarokoili, Dimitra Aloizou, Jeyanthan Charles James, Sarah Vandelanotte, Ralf Gold, Simon de Meyer, Christos Krogias

**Affiliations:** 1 Department of Neurology, St. Josef-Hospital, University Hospital of the Ruhr University Bochum, Gudrunstr 56, Bochum, 44791 Germany; 2 Department of Internal Medicine, Mt. Auburn Hospital, Harvard Medical School, Cambridge, MA USA; 3 Medical Faculty, Ruhr University Bochum, Bochum, Germany; 4 Department of Nursing, National and Kapodistrian University of Athens, Athens, Greece; 5 Laboratory for Thrombosis Research, KU Leuven, Kortrijk, Belgium; 6 Department of Neurology, Evangelisches Krankenhaus Herne, Academic Teaching Hospital of the Ruhr University Bochum, Bochum, Germany

**Keywords:** Ischemic stroke, Endovascular thrombectomy, Clot, Thrombus, Cancer-related stroke, Cancer

## Abstract

**Background:**

Cancer patients are at high risk of ischemic stroke (IS) and worse outcomes. Endovascular thrombectomy (EVT) has made the analysis of retrieved clots available. This review aimed to identify studies and case reports on IS clot analysis in cancer patients (PROSPERO Registry Number CRD420251042353).

**Methods:**

The databases of PubMed/MEDLINE and SCOPUS were systematically searched for studies and case reports involving pathological analysis of clots (from EVT or autopsy) retrieved from IS patients with cancer, following the PRISMA guidelines. Studies on children, other retrieved materials, benign tumors, or where the results of cancer patients were unavailable were excluded.

**Results:**

23 case reports and 10 studies with larger clot numbers were included. Seventeen case reports referred to tumor embolism, 3 to cancer-related thrombosis (Trousseau syndrome), 2 to mucin embolism, and 1 to post-radiation stenosis. Most clots had an atypical white appearance, with tumor cells and mucin in tumor or mucin embolism respectively, or a platelet predominance for cancer-related thrombosis. Seven of 8 larger-scale studies reported significantly higher platelet and lower red blood cell proportions for clots in cancer patients, and 5 studies showed no increased percentages of white blood cells or neutrophil extracellular traps.

**Conclusion:**

The available studies were heterogeneous, and relatively small. However, preponderance of studies shows the clots of cancer patients tend to be white and predominantly platelet-rich. Atypical appearances also occur in cases of tumor or mucin embolism. These findings bear diagnostic and therapeutic implications after IS in cancer patients, including tumor work-up in platelet-rich clots and no other identifiable cause.

**Supplementary Information:**

The online version contains supplementary material available at 10.1186/s12883-026-04739-2.

## Introduction

Ischemic stroke (IS) represents a leading cause of mortality and disability [1]. IS etiology can be hard to identify, as various pathomechanisms can underlie its occurrence, such as large artery atherosclerosis (LAA) and cardioembolism (CE) [2]. In many cases, the etiology cannot be identified and these strokes are usually classified as “embolic stroke of unknown source” (ESUS), accounting for 9–25% of all IS cases [3]. In recent years, light has been shed on the association of cancer with IS. It is estimated that 15% of cancer patients suffer from a concomitant cerebrovascular disease, while active cancer is found in approximately 10% of hospitalized IS patients; the presence of cancer is also linked to higher IS recurrence rates and overall higher mortality and functional dependency after IS [4]. It has also been shown that cancer incidence is higher after an IS, with post-IS cancer incidence being more than 3-fold compared to the general population for patients below the age of 55 [5].

A multitude of cancer-related factors elevate the risk of IS, such as vessel infiltration, radiation to head and neck, chemotherapy, operations, and frequent infections; the most commonly encountered cause, however, is a cancer-induced hypercoagulable state [4]. Cancer-associated thrombosis, also referred to as Trousseau syndrome, encompasses a wide range of thrombotic and thromboembolic complications, such thrombophlebitis, deep vein thrombosis, and non-bacterial thrombotic endocarditis (NBTE) [6]. Within this context, a new subtype of IS has recently been described, namely the cancer-related stroke (CRS). This is most commonly defined as an IS in a patient with active cancer, no other identifiable IS causes, and evidence of hypercoagulability (e.g. elevated D-dimers or history of venous thromboembolism), usually combined with embolic IS in multiple vascular territories [7]. The detection of CRS can also be tricky, as cancer may not be known at the time of IS, and entities such as NBTE complicate diagnosis, since the focus is shifted to possible infectious causes and uncovering the underlying mechanism can be delayed. A high level of alertness is required, in identifying warning signs of an occult cancer, such as laboratory marker abnormalities (e.g. anemia, inflammatory markers without clear infection), symptoms such as weight loss and nighttime fever, and risk factors from the patient history (e.g. smoking, positive family history) [4].

The acute treatment of IS consists of intravenous thrombolysis (IVT) with recombinant tissue plasminogen activator (rtPA) and endovascular thrombectomy (EVT) for large vessel occlusions (LVO) [8]. The expansion of EVT has opened a new field of study on the extracted clots. Their pathological examination has highlighted differences in the composition of clots stemming from different etiologies, while their architecture has also been shown to affect the efficacy of IVT and EVT [9]. Clots have complex and heterogeneous structures. Most commonly, they present “red” areas, which are red blood cell (RBC)- and fibrin-poor, and “white” areas, which are platelet- and fibrin-rich [10], with various coagulation factors, such von Willebrand factor, important for platelet adhesion, and factor X, which activates thrombin and induces the coagulation cascade [11]. Atypical clot presentations have also been described, stemming from rarer IS causes, and these clots may present tumor cells, or even bacteria in cases of septic embolism [12]. White blood cells (WBCs), mostly neutrophils, and neutrophil extracellular traps (NETs) are also important possible components of clots especially in inflammatory conditions, which are also known to promote thrombosis [13]. Considering that IS can be the first manifestation of an occult cancer [4], identifying the properties of cancer-borne clots can help in raising suspicion of an underlying cancerous disease, especially when combined with the aforementioned warning signs, such as laboratory abnormalities.

The aim of the present systematic review was to identify studies and case reports presenting findings on IS clot analysis in patients with cancer, in attempt to pinpoint the characteristics that can identify CRS in cases of LVO and highlight the usefulness of clot analysis in IS. To our knowledge, this is the first study with this objective (PROSPERO Registry Number CRD420251042353).

## Methods

**Fig. 1:**
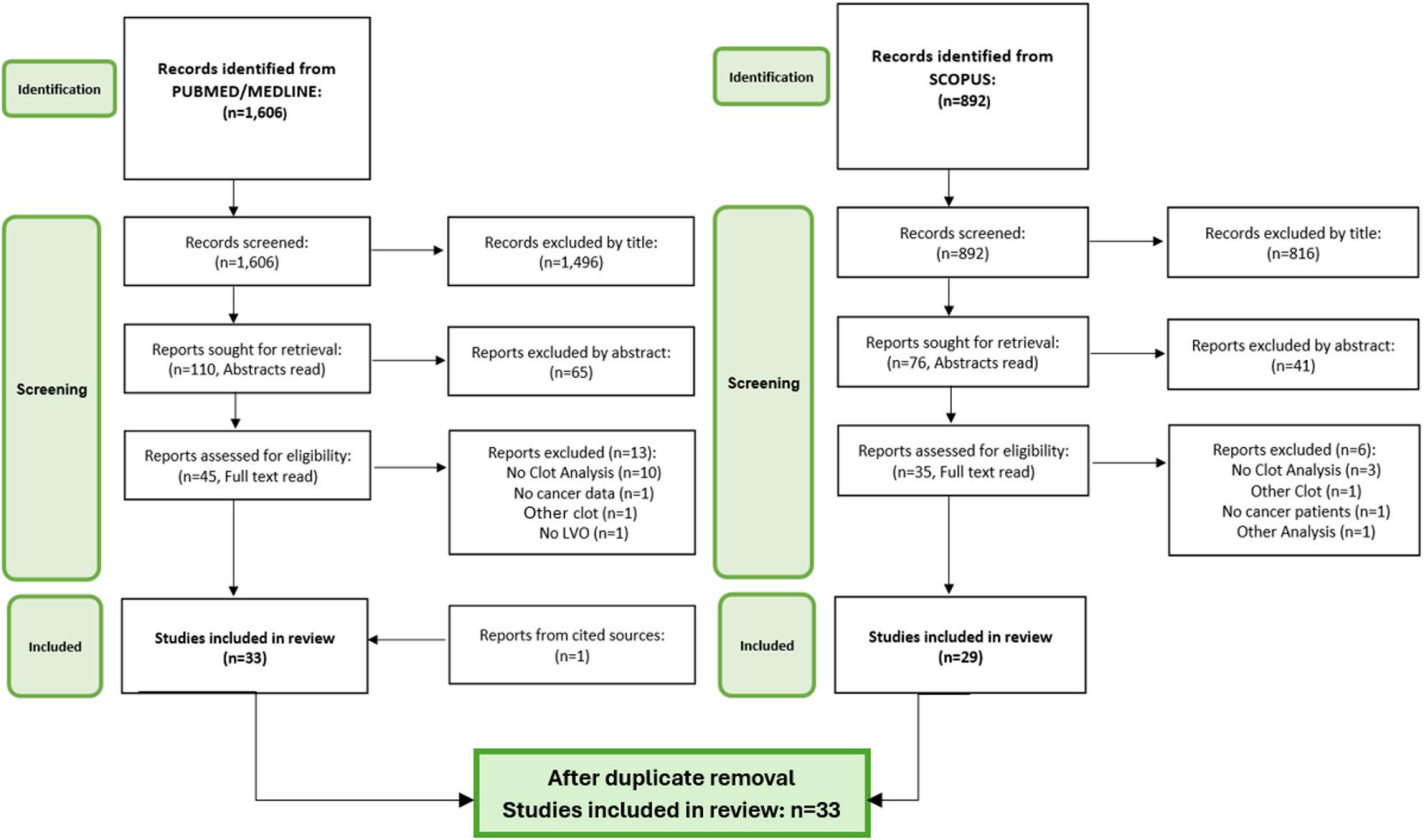
PRISMA flowchart for study inclusion, for PUBMED/MEDLINE and SCOPUS databases

The databases of Pubmed/MEDLINE and SCOPUS were searched for studies written in English, on adult IS patients, where a pathological examination of the clot material extracted from the EVT (or at autopsy for earlier studies) was undertaken, and an active cancer was already known or afterwards discovered. Studies on children (< 18 years of age) or materials that were not IS clots, or where the results for cancer patients specifically were not available, were excluded. Studies on myxoma and other non-cancerous tumors of the heart were also excluded. The words “neoplasms”, “clot”, “thrombus” and “stroke” were used as MeSH terms, with “cancer”, “malignancy”, “thrombus”, “cerebral ischemia” added as free text words, with the respective AND/OR Boolean operators. Two independent authors (A.-M.A., D.A.) performed the screening and data extraction, discrepancies were later solved by a third independent author (D.D.C.). The search was performed on the 4th of November 2024 and then actualized on the 30th of September 2025. The yielded results were assessed firstly through title and then through abstract, and those deemed potentially fitting were assessed in their full text. The references of the full texts were also assessed for any relevant studies (PRISMA Flowchart in Fig. 1). This review adheres to the PRISMA statement. Meta-analysis was not performed due to the high inter-study heterogeneity, owing to the differences in methodology and study populations; the histopathology methods from the retrieved articles were also extracted, to allow for possible comparisons, given that histopathology is mostly a semi-quantitative analysis, with high interobserver variability, limited standardization, no established cut-offs, and allowing for other technical errors. Risk of bias for the included larger scale studies was assessed with the JBI Critical Appraisal Tool for case series/studies without controls (< 2 ‘no’ answers: low, 2–3 ‘no’ answers: intermediate-low, 4–6 ‘no’ answers: intermediate-high, > 6 ‘no’ answers: high risk of bias) [14], and the Newcastle-Ottawa scale for cohort and case/control studies (8–9 stars: low, 7 − 6 stars: intermediate-low, 4–5 stars: intermediate-high, 0–3 stars: high risk of bias) [15]. Results were separated into case reports/case series and studies with larger number of clots.

## Results

### Case reports/case series

Overall, 23 case reports were identified and are summarized in Table 1.

**Table 1:** Included case reports and case series of clot analysis in cancer patients

Author, Year	Type of Cancer	Clot Composition and Methods
Baker, 2024 [16]	High grade sarcoma and further unidentified malignant foci (possible metastases, further work-up not wanted)	-Histopathology method not explicitly stated, H + E* shown, plus IH** for CD20, CD3, CD68, and CD45 -Macroscopic: white, “fatty” -Microscopic: Apoptosis and extended necrosis in the central part of the clot, malignant cells, CD 20, CD3, CD68, and CD45 negative
Bando, 2021 [17]	Lung Adenocarcinoma	-Histopathology method not explicitly stated, IH for CK7, P40, CK5/6 and TTF-1 mentioned -Macroscopic: white, fragmented -Microscopic: malignant cells, pronounced positive expression of CK7, 60% positive expression of P40, 30% positive expression of CK5/6, negative expression of TTF-1, identified as adenosquamous cell carcinoma
Conomy, 1975 [18]	Hogdkin’s lymphoma	-Histopathological method not explicitly stated -Microsocopic: fibroblastic ingrowth, with arterial wall adherence and connective tissue proliferation (post-radiation carotid stenosis)
Deck, 1978 [19]	Breast Adenocarcinoma	-Histopathological method not explicitly stated, H + E, periodic acid Schiff (PAS), alcian-blue, mucicarmine staining mentioned -Microscopic: PAS-positive, alcian-blue-positive and mucicarmine-positive material, fat droplets, no tumor cells
Fujiwara, 2023 [20]	Thyroid cancer (not otherwise described)	-H + E and vimentin staining -Macroscopic: string-form, white, mucous -Microscopic: thyroid cancer cells
Imaizumi, 1995 [21]	Lung Adenocarcinoma	-Histopathological method not explicitly stated -Microscopic: adenocarcinoma cells
Ko, 2024 [22]	Colorectal Adenocarcinoma	-Histopathological method not explicitly stated, H + E and IH for CK7, CD20, CDX-2 mentioned -Macroscopic: reddish-brown clot -Microscopic: colorectal adenocarcinoma cells
Matsumoto, 2016 [23]	Metastatic lung cancer, metastatic pancreatic cancer (2 patients)	-Histopathological method not explicitly stated, H + E mentioned -Macroscopic: white, solid -Microscopic: >90% fibrin content, no tumor cells -Comparison with 3 LAA, 2 CE, 1 ESUS clots (no statistical analysis): smaller fibrin areas, higher RBC and WBC numbers
Mitomi, 2011 [24]	Liposarcoma with cardiac metastasis	-Histopathological method not explicitly stated, H + E mentioned -Microscopic: fibrin, WBCs, tumor cells, consistent with those found in the cardiac tumor
Moriyama, 2021 [25]	Metastatic squamous cell lung cancer	-Histopathological method not explicitly stated, H + E mentioned -Microscopic: squamous cell carcinoma cells, fibrin
Nakanishi, 2021 [26]	Metastatic Renal cell carcinoma	-Histopathological method not explicitly stated, H + E mentioned -Microscopic: degenerated tumor cells, fibrin
Navi, 2009 [27]	Metastatic squamous cell lung cancer	-Histopathological method not explicitly stated, H + E mentioned -Microscopic: tumor cells within cerebral arterioles
Nicholas-Bublick, 2012 [28]	Acute promyelocytic leukemia	-Histopathological method not explicitly stated, H + E mentioned -Macroscopic: wall-adherent clot -Microscopic: blasts, fibrin
Nukata, 2023 [29]	Melanoma	-Histopathological method not explicitly stated, H + E and IH for HMB-45, melan-A, desmin, S-100, smooth muscle actin mentioned -Macroscopic: white, friable -Microscopic: malignant cells consistent with the known melanoma, small amounts of fibrin, platelets and RBCs
Passhak, 2018 [30]	Liposarcoma with cardiac metastasis	-Histopathological method not explicitly stated, H + E shown -Microscopic: tumor cells, consistent with the cells of the primary tumor
Ramachandren, 2016 [31]	Angiosarcoma of the carotid artery	-H + E, IH for vimentin, CD31, Ets-related gene, CD34, FLI-1 -Macroscopic: wall-adherent, friable clot -Microscopic: necrotic tissue with atypical epithelioid cells, positive for Vimentin, CD31, Ets-related gene, CD34 and FLI-1, consistent with malignant epithelial angiosarcoma
Ridha, 2019 [32]	Dermatofibrosarcoma protuberans, old thyroid cancer	- Histopathological method not explicitly stated, H + E and Von Kossa staining mentioned -Macroscopic: white -Microscopic: fibrinous exudate, no calcium depositions
Sun, 2022 [33]	Gastric cancer	-H + E, IH with anti-CD42b, anti-fibrinogen, anti-glycophorin A -Macroscopic: white -Microscopic: platelet-rich, erythrocyte-poor
Takasugi, 2017 [34]	Squamous cell oropharyngeal cancer	-Histopathological method not explicitly stated, H + E and Elastica van Gieson staining mentioned -Microscopic: tumor cells within cerebral arterioles
Tando, 2024 [35]	Intravascular lymphoma	-H + E, Masson trichrome staining, IH for CD3, CD5, C10, CD20, CD44, CD79a, NFκB p50, TNFα, IL-1 β, IL-6, Bcl-6, MUM1 -Macroscopic: wall-adherent, white parts -Microscopic: CD20, CD44 and TNFα positive lymphoma cells attached to the artery wall, mixed with fibrin, angiogenesis
Towfighi, 1983 [36]***	Breast cancer	-Histopathological method not explicitly stated -Microscopic: mucin and fat emboli
Wolpert, 2020 [37]	Non-small cell lung cancer	-H + E, Papanicolaou staining, IH for TTF1, pancytokeratin, CD56, synaptophysin -Microscopic: clusters of pancytokeratin, CD56- and synaptophysin-positive tumor cells with a high nucleocytoplasmic ratio, typical of a neuroendocrine carcinoma, appr. 20% of cells within the clot identified as tumor cells
Yoshikawa, 2020 [38]	Pleomorphic lung cancer	-Histopathological method not explicitly stated, H + E shown -Macroscopic: white, elastic -Microscopic: central necrosis, tumor cells consistent with the lung cancer

*H + *E*: hematoxylin and eosin staining, ** *IH* immunohistochemical staining. ***no full text available

In short, 17 reports mentioned tumor emboli, with lung cancer being the most commonly encountered cancer (6 cases), followed by sarcoma (4 cases). Cardiac metastasis and pulmonal vein infiltration were described in 4 cases each. Tumor cells of the respective cancers were identified in all cases, often entangled with fibrin fibers. The macroscopic appearance was provided in 7 cases, in 6 of which the clot was described as pale. Ten cases reported a bad prognosis, with death of the patients shortly after the tumor embolism. Mucin embolism without tumor cells was reported in 2 cases, while 3 reports with 4 patients referred to Trousseau syndrome; the clots were macroscopically white and with a platelet/fibrin predominance. In one of the reports, the clot retrieved from a venous embolism in the reported IS patients, presented a different (RBC-rich) morphology [33]. One report described carotid wall anomalies with fibroblastic ingrowth of the clot in post-cervical radiation carotid stenosis and occlusion [18].

### Studies with larger number of clots

Ten studies attempted to analyze larger numbers of clots (Table 2). No study showed a high risk of bias.

**Table 2:** Studies with analyses of larger numbers of clots

Author, Year	Study Design	Relevant Results	Risk of Bias
Cha, 2022 [39]	-Consecutive LVO patients undergoing EVT, 75 clots, 7 cancer patients -Histopathology: H + E, IH with anti-glycophorin A (heat-induced epitope retrieval), anti-CD42b, anti-fibrinogen, and neutrophil elastase, automated region-of-interest-based image analysis and percentage of pixel density calculation -Division and comparison of high vs. low neutrophil content clots based on median value of the neutrophil fraction	-CE more frequent in the high-neutrophil group, LAA more frequent in the low-neutrophil group (*p* = 0.035) -Association of high neutrophil counts with RBC-rich clots -6/7 cancer patient clots with low neutrophil content (very small number, no statistical difference, *p* = 0.121)	Low
Fu, 2020 [40]	-Consecutive LVO patients undergoing EVT,152 clots (19 CRS, 107 CE, 26 LAA clots) -Histopathology: H + E, IH with anti-CD42b, application of the Orbit Image Analysis software, automated region-of-interest-based image analysis and percentage of pixel density calculation -Comparison of clot content within the 3 groups, regression analysis and AUC-ROC/ Youden’s index analysis of fibrin percentage in differentiating AC clots	-CRS clots significantly richer in fibrin/platelets (85.7% vs. 43.9% and 42.5%), poorer in RBC (8.1% vs. 52.2% and 51.7%; %; *p* < 0.001) and WBC (1.9% vs. 3.7% and 3.1%, *p* = 0.02) -Fibrin/platelet proportion of ≥ 65%: AUC 0.84 (*p* < 0.001) for CRS identification -12/19 CRS patients with adenocarcinomas, these clots showing significantly larger CD42b positive areas	Low
Fu, 2025 [41]	-Consecutive LVO patients undergoing EVT, 49 active cancer, 23 non-active cancer, 316 no cancer clots -Histopathology: H + E, application of the Orbit Image Analysis software, automated region-of-interest-based image analysis and percentage of pixel density calculation -Comparison of content within the 3 groups, regression analysis and AUC-ROC/Youden’s index of fibrin and platelet percentage in predicting stroke recurrence or mortality (primary outcome)	-Active cancer clots significantly richer in platelets and fibrin (69.3% vs. 47.6% and 45.8% *p* < 0.001) -Higher platelet/fibrin proportion associated with stroke recurrence or mortality within 3 months (aOR 1.03, *p* = 0.048), within the active cancer group - Fibrin/platelet proportion of ≥ 79.4%: AUC 0.73 (*p* = 0.006) for predicting stroke recurrence or mortality in active cancer, 24/49 of active cancer patients with a fibrin/platelet proportion of ≥ 79.4% -Clot composition in patients without active cancer (*n* = 339) not significantly associated with specific primary or secondary outcomes after multivariable adjustments -31/50 active cancer patients with adenocarcinomas	Low
Genchi, 2021 [42]	-Consecutive LVO patients undergoing EVT, 80 clots, examined for neutrophils and NETs -Histopathology: IH for citrullinated histone H3 and myeloperoxidase (MPO), automated NET calculation with the Aperio^®^ Color Deconvolution Algorithm on ImageScope, as percentage of histone H3 on the total thrombus area, automated neutrophil calculation (MPO positive cells) with Mumford- Shah segmentation algorithm on Orbit Image -Comparisons between different stroke etiologies, regression analysis for median of clot NETs	-CE clots richer in NETs compared to LAA (*p* = 0.04) -Cancer not significant for median NET in multivariate analysis [β coefficient, CI: 2.76 (6.09 to 0.57), *p* = 0.1]	Low
Heo, 2023 [43]	-70,561 images from 182 patients (119 patients in internal datasets, 63 in external datasets) from a registry of consecutive LVO patients undergoing EVT, provided for machine learning models, 5/23 patients with active cancer for the internal validation, 12/63 patients with cancer for the external validation -Histopathology: IH with anti-CD42b, rabbit polyclonal antifibrinogen, and rabbit monoclonal anti–glycophorin A, automated ROI-based image analysis (https://github.com/jnheo-md/aria) with investigator confirmation, use of the then color-processed images for the machine learning models -Comparison between groups (active cancer vs. other determined etiology strokes), AUC-ROC/Youden’s index for platelet, fibrinogen and glycophorin A models in prediction of active cancer	-Cancer clots significantly richer in platelets (38.2% vs. 17.2%, *p* < 0.001), poorer in RBCs (15.9% vs. 48%, *p* < 0.001) and fibrinogen (35.4% vs. 39.7%, *p* = 0.033) compared to patients with other determined etiology strokes -Platelet model: AUC > 0.94 in identifying active cancer patients	Low
Ikeda, 2023 [44]	-Consecutive LVO patients with any history of cancer undergoing EVT, 12 white (fibrin/platelet percentage ≥ 80%) and 11 red (fibrin/platelet percentage < 80%) clots -Histopathology: H + E, quantitative image analysis with the ImageJ software -Comparisons between groups, regression analysis for laboratory parameter association with white clots	-Active cancer more common in white clots (11/12 vs. 4/11, *p* = 0.0094)	Low
Magami, 2024 [45]	-108 LVO patients undergoing EVT/112 procedures (unclear if consecutive), 6 CRS and 92 non-CRS clots (CRS definition incomplete) -Histopathology: H + E, no further details on the analysis -No statistical analysis regarding clots	-1/6 CRS clots vs. 65/92 non-CRS clots RBC-rich (no statistical comparison) -4/7 cancer patients with adenocarcinomas	Intermediate-low
Park, 2019 [46]	-Consecutive patients with LVO and cancer, 16 active cancer, 16 inactive cancer, 16 no cancer clots (propensity score matched) -Histopathology: H + E, IH with rabbit monoclonal anti-CD42b, antifibrinogen, anti–glycophorin A, mouse monoclonal anti-MPO, rabbit polyclonal anti–histone H3, semiautomated quantitative image analysis with the ImageJ software and percentage of pixel density calculation -Comparison between groups and one-way ANOVA for analysis of the relationship between neutrophils and NET activity	-Active cancer clots significantly richer in platelets (43.2% vs. 12.9% and 14.1%, *p* < 0.001) and poorer in RBCs (3.4% vs. 43.5% and 40.7%, *p* < 0.001) -No statistical difference in fibrin/fibrinogen (28.6% vs. 37% and 26.6%, *p* = 0.6), WBCs (80 vs. 441.4 vs. 640.0, *p* = 0.088) and NETs (1.8% vs. 2.8% and 1.5%, *p* = 0.33)	Low
Woock, 2025 [47]	-Case/control study on LVO patients from the RESTORE registry, 64 cancer patients and 64 age- and sex-matched controls (95 cancer clots, 100 non-cancer clots) -Histopathology: H + E, Martius Scarlett Blue staining, IH for von Willebrand factor (vWF), CD42b, citrullinated histone H3, machine-learning image analysis with the Orbit software, calculation of each component area with multiplication of component percent by the relevant extracted clot area - Comparison between the 2 groups	-No significant differences in RBC, platelets (and CD42b positivity), fibrin, CD3 positivity percentages -Cancer clots had a collagen percent of ≥ 1% significantly more often (16/64 vs. 3/64, *p* = 0.002), positive predictive value of 84% -Active cancer clots had a significantly higher content of vWF (median 26 [IQR13-38]% vs. 10 [4–18]%, *p* < 0.0001) and NETs (H3Cit) (median 0.11% [IQR0.02–0.46] vs. 0.05% [0.00–0.28] *p* = 0.027), after correction for multiple comparisons significance for NETs lost, for fibrin and collagen remained	Low
Yoo, 2023 [48]	-Consecutive LVO patients undergoing EVT, 23 active cancer (no other identifiable IS causes) and 23 control clots (propensity score matched) -Histopathology: H + E, IH with anti-glycophorin A, anti-CD42b, anti-neutrophil elastase, anti-CD68, anti-histone H3, anti-fibrinogen, anti-thrombin, anti-CD142, anti-factor X/Xa, anti-factor Xia, anti-factor XII, anti-factor XIII, semiautomated image analysis with opensource software automated region-of-interest–based image analysis by blinded investigators, calculation of component percentage as area fraction of pixels over a predefined density threshold, equivalent to 160 in ImageJ software, to the total thrombus area -Comparison between the 2 groups	-Active cancer clots significantly richer in platelets (51.3% vs. 9.5% *p* < 0.001), thrombin (26.2% vs. 4.5% *p* < 0.001) and tissue factor (0.60% vs. 0.37%, *p* = 0.024), and poorer in RBCs (4.2% vs. 34.4% *p* < 0.001), neutrophils (1.2% vs. 3.8%, *p* = 0.021), NETs (1.3% vs. 2.3%, *p* = 0.047), and factor X (1.25% vs. 2.33%, *p* = 0.034) -Positive correlation between thrombin and platelets only in the cancer group (*r* = 0.666; *p* = 0.001), negative correlation between thrombin and RBCs only in the control group (*r* = − 0.438; *p* = 0.036)	Low

For platelet/fibrin and RBC proportions, 8 studies were available. Despite heterogeneous designs, 7 studies reported a platelet predominance in the cancer groups compared to other IS etiologies [40, 41, 43–46, 48]; only one study showed similar proportions of RBCs, platelet, and fibrin between cancer and non-cancer clots [47]. Two studies provided evidence that a platelet/fibrin predominance in the retrieved clots could identify CRS with good statistical results [40, 43].

For WBC, 2 studies reported lower or similar WBC/neutrophil and NET percentages in the clots of cancer and non-cancer patients [46, 48]. One study reported a numerical overrepresentation of cancer patients in the low-neutrophil vs. the high-neutrophil group (6 vs. 1, no statistical significance) [39], and another study could not associate cancer with NETs in their multivariate analysis [42]. Only one study contradicted this, showing that cancer clots presented a significantly higher percentage of NETs, though after correction for multiple comparisons, the significance was lost [47].

Regarding coagulation factors, one study reported higher expression of thrombin and tissue factor, and lower factor X expression, with comparable levels of factors I, XII and XIII in cancer and non-cancer clots; thrombin and platelets were also only correlated positively in the cancer group [48]. A second study reported significantly higher von Willebrand Factor (vWF) percentages in cancer clots [47].

## Discussion

Summing up the results of our review on the available case reports and larger studies, cancer patients tend to demonstrate macroscopically pale, and microscopically platelet- and fibrin-rich clots. Rarer occasions with tumor cell and mucin emboli were also reported, with an atypical macroscopical appearance as well.

The platelet and fibrin predominance of cancer-related clots can be explained through direct cancer-induced platelet activation and aggregation, a well-established phenomenon in cancer-related thrombosis, which promotes tumor survival and metastasis [49]. Thrombocytosis can also be induced by a tumor-driven cytokine production, leading to the creation of platelet aggregates, which protect tumor cells from natural killer cells [48]. Additionally, cancer cells are also capable of directly producing thrombin, the strongest platelet activator, or producing tissue factor, which in turn enhances thrombin production [48, 49]. VWF, with its known thrombogenic potential and platelet aggregation ability, has also been reported as elevated in serum, tissues, and clots of cancer patients [47], also thought to facilitate cancer spread via induction of cancer-platelet interaction. The promotion of cancer survival through platelet activation and subsequent metastasis can also explain the link between cancer-related thrombosis in advanced disease with stroke recurrency and higher mortality [41].

The finding that clots in cancer patients tend to be platelet-rich is concordant with the findings of Cha et al., showing more cancer patients in the low-neutrophil group, where platelet-rich clots were also more prominent [39]. However, the results regarding WBCs and NETs were not as clear as RBCs and platelets. WBCs and NETs have an established role in cancer-related thrombosis; cancer cells can induce tissue factor expression on monocytes or its release in extracellular vesicles, and NETs, produced from activated neutrophils, can capture circulating platelets and extracellular vesicles, and trigger thrombosis [50]. Despite this, the few available studies could not reveal higher WBC or NET percentages in the clots of cancer patients, raising doubt concerning their involvement in large-scale arterial events. Indeed, NETs could be identified in autopsy samples of various arterial microthrombi of cancer patients without large-vessel pathology [51]. It is possible, that local endothelial damage in cancer, as showcased for example through elevated PAI-1 (plasminogen activator inhibitor-1) levels in cancer patients [50], leads to the initial local recruitment of WBCs, the excretion of NETs, and then to an exaggerated platelet recruitment, giving way to the platelet predominance, without an obvious elevation of WBCs and NETs in the thrombus itself.

Following this line of thought, a case report showed different clot morphologies in arterial and venous clots in the same cancer patient [33], highlighting the possibility that arterial and venous thromboses may have different underlying mechanisms in cancer. This could also explain why anticoagulants such as low molecular weight heparins and factor Xa inhibitors, with proven efficacy in venous thromboembolism, have not demonstrated a similar efficacy in preventing recurrent stroke in CRS patients [7], in accordance to our results of platelet-predominance in cancer-related clots. Congruently, Yoo et al. reported lower Factor X expression in clots retrieved from cancer patients [48], which could hint to factor Xa-antagonists not providing any anticoagulation benefits in CRS. It should be mentioned however, that IS in cancer patients represent a particular challenge, since concomitant pathologies such as atrial fibrillation or carotid stenosis often conceal cancer-induced mechanisms, which are not properly identified. This also impacts treatment decisions, since the focus is shifted onto comorbidities, and not on cancer treatment or detection thereof. IS documentation in cancer patients also leads to discrepancies and difficulties in interpreting retrospective studies, as CRS can either be classified as ESUS, or as IS of other identifiable source, and cases may be then lost in the analysis.

In this sense, more studies exploring CRS clots exclusively are needed, since not every cancer patient with IS fulfills the criteria of CRS. For instance, Park et al. showed that despite their active cancer group as a whole demonstrating higher platelet and lower RBC percentages, active cancer patients with other determinable etiologies had higher RBC fractions, comparable to clots from non-active cancer patients; on the contrary, patients with cancer-related coagulable conditions, for example NBTE, and undetermined etiologies, showed even higher platelet counts [46]. As such, it seems that a white, platelet-rich clot bears ties to the underlying cancer and identifying the clot traits associated with cancer is particularly important in cases of occult cancer, where the failure to identify traditional stroke risk factors should prompt further investigation. The pathological examination of clots is not a standard in most hospitals, though an atypical appearance can even be detected during the EVT; an interesting study evaluated only the macroscopic aspects of clots and showed that in the white clot group, atypical etiologies were significantly more common, with the difference increasing considerably when only advanced cancer was considered [52]. Our results suggest that an atypical thrombus morphology with platelet predominance, in the absence of a clear IS etiology should prompt further diagnostic work-up to exclude an occult cancer, such as advanced imaging or further laboratory examinations. This is of utmost importance, since the diagnosis of such a disease will have important therapeutic implications for the patient. Additionally, the detection of tumor embolism, an otherwise rare entity [37], also carries therapeutic consequences even in the setting of known cancer, as cases of brain metastases occurring in the infarcted area have been reported [53, 54], and heightened vigilance is advised. Furthermore, the effect of clot composition on recanalization success also needs to be discussed. It is a well-established fact, that cancer patients with IS, and CRS patients in particular, have an overall worse prognosis in terms of long-term functional outcome and mortality [55], although important differences in safety and efficacy parameters for IVT [56] and EVT [57, 58] have not been documented between patients with and without cancer. For IVT, platelet and fibrin-rich clots tend to be more compact, allowing for less rtPA penetration in the clot; as such, RBC-rich clots are more responsive to IVT [9]. Though studies on clots from cancer patients and IVT efficacy are lacking, it can be assumed that the platelet predominance predisposes to reduced IVT efficacy rates. For EVT, overall recanalization rates, assessed with Thrombolysis in Cerebral Ischemia (TICI) scores, have been reported as comparable between patients with and without cancer [55]. Nevertheless, clot composition may still affect the speed and effectiveness of EVT, in terms of first pass effect (FPE) and recanalization times, which have consistently been linked to better outcomes [59, 60]. Platelet-rich clots demonstrate increased stiffness [61], and the studies of Maekawa et al. and Karimian-Jazi et al. showed that RBC-rich clots required fewer passes and less time to extract, while they were also more responsive to contact aspiration [62, 63]. Similarly, in the study by Noguiera et al. (2024), platelet-rich clots required a higher number of passes, despite the rest of the EVT parameters not showing a clear association with clot composition [64]. As such, since cancer patients tend to form more platelet-rich clots, they might be susceptible to worse outcomes regardless of the final TICI score.

In this regard, Jeon et al. reported that between CRS patients, contact aspiration showed significantly higher FPE rates than stent retriever EVT, with similar complication rates [68]. Similarly, Yun et al. [65] reported no statistically significant differences in FPE or other EVT parameters in CRS patients comparing stent retriever, contact aspiration, and the combined method (with similar rates of white and red clots in all groups), although the absolute number of patients with FPE was higher in the contact aspiration and the combined groups (appr. 35% compared to 17% in the stent retriever group); the dual layered stent retrieved produced consistently better results [65]. Finally, Ozaki et al. showed worse recanalization rates with the use of small caliber contact aspiration in cancer patients, with no notable differences regarding direct aspiration, stent retriever, or combined method compared to non-cancer patients [66]. These findings could carry important practical implications when a patient with a suspected CRS is selected to undergo EVT, as selecting the optimal EVT method could lead to faster and easier recanalization.

Woock et al. also provided another interesting finding, regarding collagen. Clots from cancer patients were significantly more likely to have a high collagen content (≥ 1%), while the vast majority of patients with high collagen (86%) had advanced disease, mostly metastatic [47]. The remodeling of the extracellular matrix plays an important role in cancer proliferation and metastasis, with cancer-associated fibroblasts and the collagen types they produce driving this process [67]. Collagen may also alter the stiffness of the clot, potentially impeding recanalization.

Finally, the large heterogeneity of the available studies needs to be discussed. This is attributable to the fact that EVT has only expanded in the last years, and pathological analysis is not of standard in most hospitals. Combined with the fact that cancer patient numbers tend to be small, the number of publications is still limited, so no homogenous protocols are largely available to be followed, and the ones available have applied different histopathology and image analysis methods and software, allowing for different errors. Additionally, several studies had a different focus, for example identifying differences in clots of various syntheses, where cancer was in this case an examined parameter among others. In order to be able to exact more reliable results in the future, we suggest that undertaken endeavors try to follow methodologies of existing studies, for instance directly comparing cancer and non-cancer patient clots, so as to facilitate the extraction of accurate conclusions.

## Conclusions

Cancer-related clots seem to bear a unique appearance, being macroscopically whiter, and with a platelet predominance. In rarer cases, tumor cells or mucin may be pathologically identified, providing a direct link between cancer and IS. Considering how no efficient prophylaxis for IS in CRS is known, further clot analysis studies are needed, to shed light on clot formation in active cancer, and guide therapeutic decision making.

## Supplementary Information

Supplementary Material 1.

## Data Availability

Data sharing is not applicable to this article as no datasets were generated or analyzed during the current study.

## References

[1] Wafa HA, Wolfe CDA, Emmett E, Roth GA, Johnson CO, Wang Y. Burden of stroke in europe: Thirty-Year projections of Incidence, Prevalence, Deaths, and Disability-Adjusted life years. Stroke. 2020;51(8):2418–27. 32646325 10.1161/STROKEAHA.120.029606 PMC7382540

[2] Adams HP Jr., Bendixen BH, Kappelle LJ, Biller J, Love BB, Gordon DL, et al. Classification of subtype of acute ischemic stroke. Definitions for use in a multicenter clinical trial. TOAST. Trial of org 10172 in acute stroke treatment. Stroke. 1993;24(1):35–41. 7678184 10.1161/01.str.24.1.35

[3] Hart RG, Catanese L, Perera KS, Ntaios G, Connolly SJ. Embolic stroke of undetermined source: A systematic review and clinical update. Stroke. 2017;48(4):867–72. 28265016 10.1161/STROKEAHA.116.016414

[4] Dardiotis E, Aloizou AM, Markoula S, Siokas V, Tsarouhas K, Tzanakakis G, et al. Cancer-associated stroke: Pathophysiology, detection and management (Review). Int J Oncol. 2019;54(3):779–96. 30628661 10.3892/ijo.2019.4669 PMC6365034

[5] Vaz CG, Rodrigues J, Pereira D, Matos I, Oliveira C, Bento MJ, et al. The crosstalk between stroke and cancer: incidence of cancer after a first-ever cerebrovascular event in a population-based study. Eur Stroke J. 2023;8(3):792–801. 37317526 10.1177/23969873231181628 PMC10472965

[6] Ikushima S, Ono R, Fukuda K, Sakayori M, Awano N, Kondo K. Trousseau’s syndrome: cancer-associated thrombosis. Jpn J Clin Oncol. 2016;46(3):204–8. 26546690 10.1093/jjco/hyv165

[7] Aloizou AM, Palaiodimou L, Aloizou D, Dardiotis E, Gold R, Tsivgoulis G, et al. Acute reperfusion treatment and secondary prevention of cancer-related stroke: comprehensive overview and proposal of clinical algorithm. Ther Adv Neurol Disord. 2023;16:17562864231180717. 37342814 10.1177/17562864231180717 PMC10278431

[8] Aloizou AM, Siokas V, Mentis AA, Dastamani M, Sokratous M, Xiromerisiou G, et al. Advancements in the treatment of cerebrovascular complications of cancer. Curr Treat Options Neurol. 2020;22. 10.1007/s11940-020-00624-6.

[9] Jolugbo P, Ariens RAS. Thrombus composition and efficacy of thrombolysis and thrombectomy in acute ischemic stroke. Stroke. 2021;52(3):1131–42. 33563020 10.1161/STROKEAHA.120.032810 PMC7610448

[10] Staessens S, Denorme F, Francois O, Desender L, Dewaele T, Vanacker P, et al. Structural analysis of ischemic stroke thrombi: histological indications for therapy resistance. Haematologica. 2020;105(2):498–507. 31048352 10.3324/haematol.2019.219881 PMC7012484

[11] Mast AE. Tissue factor pathway inhibitor: multiple anticoagulant activities for a single protein. Arterioscler Thromb Vasc Biol. 2016;36(1):9–14. 26603155 10.1161/ATVBAHA.115.305996 PMC4690769

[12] Aspegren O, Staessens S, Vandelanotte S, Desender L, Cordonnier C, Puy L, et al. Unusual histopathological findings in mechanically removed stroke Thrombi - A multicenter experience. Front Neurol. 2022;13:846293. 35665052 10.3389/fneur.2022.846293 PMC9157388

[13] Zhou Y, Xu Z, Liu Z. Impact of neutrophil extracellular traps on thrombosis formation: new findings and future perspective. Front Cell Infect Microbiol. 2022;12:910908. 35711663 10.3389/fcimb.2022.910908 PMC9195303

[14] Munn Z, Barker TH, Moola S, Tufanaru C, Stern C, McArthur A, et al. Methodological quality of case series studies: an introduction to the JBI critical appraisal tool. JBI Evid Synth. 2020;18(10):2127–33. 33038125 10.11124/JBISRIR-D-19-00099

[15] Stang A. Critical evaluation of the Newcastle-Ottawa scale for the assessment of the quality of nonrandomized studies in meta-analyses. Eur J Epidemiol. 2010;25(9):603–5. 20652370 10.1007/s10654-010-9491-z

[16] Baker R, Bakali Z, Crocker JS, Mowla A, Smith M, Grossman A, et al. Tumor embolic stroke: the importance of pathological assessment of clots after thrombectomy. J Clin Med. 2024;13(7):1834. 10.3390/jcm13071834. 10.3390/jcm13071834 PMC11012646 38610599

[17] Bando t, Ueno Y, Kuroyama T, Shimo D, Mikami K, Hori S, et al. Histopathological diagnosis of clot tissues collected by mechanical thrombectomy provides Understanding of cerebral infarction pathology in cancer associated thrombosis: a case report. Interdiscip Neurosurg. 2021;25. 10.1016/j.inat.2021.101211.

[18] Conomy JP, Kellermeyer RW. Delayed cerebrovascular consequences of therapeutic radiation. A clinicopathologic study of a stroke associated with radiation-related carotid arteriopathy. Cancer. 1975;36(5):1702–8. 1192359 10.1002/1097-0142(197511)36:5<1702::aid-cncr2820360525>3.0.co;2-v

[19] Deck JH, Lee MA. Mucin embolism to cerebral arteries: a fatal complication of carcinoma of the breast. Can J Neurol Sci. 1978;5(3):327–30. 212169 10.1017/s0317167100024434

[20] Fujiwara Y, Hayashi K, Shibata Y, Furuta T, Yamasaki T, Yamamoto K, et al. Cerebral tumor embolism from thyroid cancer treated by mechanical thrombectomy: illustrative case. J Neurosurg Case Lessons. 2023;5(5). 10.1159/000196412. 10.3171/CASE22293 PMC10550714 36718865

[21] Imaizumi K, Murate T, Ohno J, Shimokata K. Cerebral infarction due to a spontaneous tumor embolus from lung cancer. Respiration. 1995;62(3):155–6. 7569337 10.1159/000196412

[22] Ko LY, Kok VC, Tang CH, Lee CK, Yen PS. Successful recanalization and neurological restoration in cancerous embolic cerebral infarction via endovascular Stent-Retriever embolectomy. Onco Targets Ther. 2024;17:573–8. 39055326 10.2147/OTT.S470306 PMC11269397

[23] Matsumoto N, Fukuda H, Handa A, Kawasaki T, Kurosaki Y, Chin M, et al. Histological examination of Trousseau Syndrome-Related thrombus retrieved through acute endovascular thrombectomy: report of 2 cases. J Stroke Cerebrovasc Dis. 2016;25(12):e227–30. 27720526 10.1016/j.jstrokecerebrovasdis.2016.08.041

[24] Mitomi M, Kimura K, Iguchi Y, Hayashida A, Nishimura H, Irei I, et al. A case of stroke due to tumor emboli associated with metastatic cardiac liposarcoma. Intern Med. 2011;50(14):1489–91. 21757835 10.2169/internalmedicine.50.5071

[25] Moriyama T, Sugiura Y, Hayashi Y, Kinoshita F, Yamamura R, Moriya M, et al. Mechanical thrombectomy for acute middle cerebral artery occlusion caused by tumor embolism: A case report. J Neuroendovasc Ther. 2021;15(1):52–7. 37503456 10.5797/jnet.cr.2020-0022 PMC10370610

[26] Nakanishi K, Kawano H, Yamagishi Y, Kamma H, Shiokawa Y, Hirano T. Tumor cells detected in retrieved thrombus: Cancer-associated stroke. Intern Med. 2021;60(15):2491–4. 33678737 10.2169/internalmedicine.6201-20 PMC8381188

[27] Navi BB, Kawaguchi K, Hriljac I, Lavi E, DeAngelis LM, Jamieson DG. Multifocal stroke from tumor emboli. Arch Neurol. 2009;66(9):1174–5. 19752313 10.1001/archneurol.2009.172

[28] Nicholas-Bublick S, Irlam JH, Tietjen G. A malignant case of acute promyelocytic leukemia with occlusion of carotid artery by tumor thrombus. J Stroke Cerebrovasc Dis. 2012;21(4):325–6. 22182759 10.1016/j.jstrokecerebrovasdis.2010.08.003

[29] Nukata R, Ikeda H, Akaike N, Fujiwara T, Yamashita H, Uezato M, et al. White Embolus-induced Basilar artery occlusion due to pulmonary vein invasion of a metastasis of a malignant melanoma. Intern Med. 2023;62(19):2889–93. 36823083 10.2169/internalmedicine.1269-22 PMC10602821

[30] Passhak M, Amsalem Y, Vlodavsky E, Varaganov I, Bar-Sela G. Cerebral liposarcoma embolus from heart metastasis successfully treated by endovascular extraction followed by cardiac surgery. Vasc Endovascular Surg. 2018;52(8):653–7. 29940813 10.1177/1538574418783527

[31] Ramachandren TK, Venkataraman K, Hussey K, Ferguson L. Metastatic angiosarcoma presenting as ischemic anterior circulation stroke. Ann Vasc Surg. 2016;31:e2097–9. 10.1016/j.avsg.2015.08.025 26620379

[32] Ridha M, Malek A, Arkun K, Leung LY. The utility of histopathologic characteristics of thrombi in large vessel occlusions. Neurol Clin Pract. 2019;9(6):481–3. 32042484 10.1212/CPJ.0000000000000630 PMC6927435

[33] Sun YE, Na HK, Kwak S, Kim YD, Nam HS, Heo JH. Different thrombus histology in a cancer patient with deep vein thrombosis and recurrent strokes. J Stroke. 2022;24(2):300–2. 35677986 10.5853/jos.2021.04140 PMC9194546

[34] Takasugi J, Sakaguchi M, Oyama N, Gon Y, Terasaki Y, Sasaki T, et al. Recurrent stroke due to metastatic pulmonary tumor emboli as an important clinical entity. J Stroke Cerebrovasc Dis. 2017;26(6):e108–10. 28366663 10.1016/j.jstrokecerebrovasdis.2017.03.012

[35] Tando S, Kimura T, Mizuhara R, Yuki N, Yoshioka A, Takahashi H, et al. An autopsy case of intravascular large B-cell lymphoma showing a rapid transition to embolic strokes with occlusion of the major cerebral arteries. Neuropathology. 2024;44(2):135–46. 37559506 10.1111/neup.12940

[36] Towfighi J, Simmonds MA, Davidson EA. Mucin and fat emboli in mucinous carcinomas. Cause of hemorrhagic cerebral infarcts. Arch Pathol Lab Med. 1983;107(12):646–9. 6314926

[37] Wolpert F, Kulcsar Z, Hansel M, Rushing E, Seystahl K, Schweizer J, et al. Embolization of tumor cells is rare in patients with systemic cancer and cerebral large vessel occlusion. Eur J Neurol. 2020;27(10):2041–6. 32492228 10.1111/ene.14372

[38] Yoshikawa S, Kamide T, Kasakura S, Arai N, Osada T, Mouri A, et al. A case of cerebral infarction due to pleomorphic carcinoma of the lung. Surg Neurol Int. 2020;11:217. 32874720 10.25259/SNI_37_2020 PMC7451184

[39] Cha MJ, Ha J, Lee H, Kwon I, Kim S, Kim YD, et al. Neutrophil recruitment in arterial thrombus and characteristics of stroke patients with Neutrophil-Rich thrombus. Yonsei Med J. 2022;63(11):1016–26. 36303310 10.3349/ymj.2022.0328 PMC9629897

[40] Fu CH, Chen CH, Lin YH, Lee CW, Tsai LK, Tang SC, et al. Fibrin and Platelet-Rich composition in retrieved thrombi hallmarks stroke with active cancer. Stroke. 2020;51(12):3723–7. 33138690 10.1161/STROKEAHA.120.032069

[41] Fu CH, Chen CH, Lin YH, Lee CW, Tsai LK, Tang SC, et al. High fibrin and platelet clot predicts stroke recurrence or mortality after thrombectomy in patients with active cancer. J Neurointerv Surg. 2025;17(11):1189–1194. 10.1136/jnis-2024-022033. 10.1136/jnis-2024-022033 39216988

[42] Genchi A, Semerano A, Gullotta GS, Strambo D, Schwarz G, Bergamaschi A, et al. Cerebral thrombi of cardioembolic etiology have an increased content of neutrophil extracellular traps. J Neurol Sci. 2021;423:117355. 33647733 10.1016/j.jns.2021.117355

[43] Heo J, Lee H, Seog Y, Kim S, Baek JH, Park H, et al. Cancer prediction with machine learning of thrombi from thrombectomy in stroke: multicenter development and validation. Stroke. 2023;54(8):2105–13. 37462056 10.1161/STROKEAHA.123.043127

[44] Ikeda H, Ishibashi R, Kinosada M, Uezato M, Hata H, Kaneko R, et al. Factors related to white thrombi in acute ischemic stroke in cancer patients. Neuroradiol J. 2023;36(4):453–9. 36607169 10.1177/19714009221150856 PMC10588610

[45] Magami S, Yoshida K, Nakao Y, Oishi H, Yamamoto T. A Single-Center experience of mechanical thrombectomy for Cancer-Associated ischemic stroke. J Neuroendovasc Ther. 2024;18(2):37–46. 38384394 10.5797/jnet.oa.2023-0067 PMC10878738

[46] Park H, Kim J, Ha J, Hwang IG, Song TJ, Yoo J, et al. Histological features of intracranial thrombi in stroke patients with cancer. Ann Neurol. 2019;86(1):143–9. 31025392 10.1002/ana.25495

[47] Woock M, Rossi R, Jabrah D, Douglas A, Redfors P, Nordanstig A, et al. Clot signature in patients with large vessel occlusion stroke and concomitant active cancer. Eur J Neurol. 2025;32(1):e70037. 39760182 10.1111/ene.70037 PMC11702498

[48] Yoo J, Kwon I, Kim S, Kim HM, Kim YD, Nam HS, et al. Coagulation factor expression and composition of arterial thrombi in Cancer-Associated stroke. Stroke. 2023;54(12):2981–9. 37886852 10.1161/STROKEAHA.123.044910

[49] Heo JH, Yun J, Kim KH, Jung JW, Yoo J, Kim YD, et al. Cancer-Associated stroke: thrombosis Mechanism, Diagnosis, Outcome, and therapeutic strategies. J Stroke. 2024;26(2):164–78. 38836266 10.5853/jos.2023.03279 PMC11164583

[50] Tatsumi K. The pathogenesis of cancer-associated thrombosis. Int J Hematol. 2024;119(5):495–504. 38421488 10.1007/s12185-024-03735-x

[51] Thalin C, Demers M, Blomgren B, Wong SL, von Arbin M, von Heijne A, et al. NETosis promotes cancer-associated arterial microthrombosis presenting as ischemic stroke with troponin elevation. Thromb Res. 2016;139:56–64. 26916297 10.1016/j.thromres.2016.01.009 PMC4769435

[52] Sgreccia A, Duchmann Z, Desilles JP, Lapergue B, Labreuche J, Kyheng M, et al. Association between acute ischemic stroke etiology and macroscopic aspect of retrieved clots: is a clot’s color a warning light for underlying pathologies? J Neurointerv Surg. 2019;11(12):1197–200. 31053576 10.1136/neurintsurg-2019-014905

[53] Ben-Hur T, Siegal T. Brainstem infarction due to tumor embolus. Cerebrovasc Dis. 1996;6:52–3.

[54] Cho Y, Hida Y, Kaga K, Kato H, Iizuka M, Kondo S. Brain metastases secondary to tumor emboli from primary lung cancer during lobectomy. Ann Thorac Surg. 2008;86(1):312–3. 18573452 10.1016/j.athoracsur.2008.01.029

[55] Aloizou AM, Richter D, Charles James J, Lukas C, Gold R, Krogias C. Mechanical thrombectomy for acute ischemic stroke in patients with malignancy: a systematic review. J Clin Med. 2022;11(16). 10.3390/jcm11164696. PMID: 36012933. 10.3390/jcm11164696 PMC9410462 36012933

[56] Mosconi MG, Capponi A, Paciaroni M. Systemic thrombolysis in patients with acute stroke and active cancer: a systematic review and meta-analysis. Intern Emerg Med. 2023;18(6):1843–50. 37337013 10.1007/s11739-023-03312-w

[57] Eun MY, Jeon ET, Seo KD, Lee D, Jung JM. Reperfusion therapy in acute ischemic stroke with active cancer: A Meta-Analysis aided by machine learning. J Stroke Cerebrovasc Dis. 2021;30(6):105742. 33780696 10.1016/j.jstrokecerebrovasdis.2021.105742

[58] Caimano D, Letteri F, Capasso F, Limbucci N, Nencini P, Sarti C, et al. Endovascular treatment in patients with acute ischemic stroke and cancer: systematic review and meta-analysis. Eur Stroke J. 2022;7(3):204–11. 36082266 10.1177/23969873221100897 PMC9446332

[59] Zaidat OO, Castonguay AC, Linfante I, Gupta R, Martin CO, Holloway WE, et al. First pass effect: A new measure for stroke thrombectomy devices. Stroke. 2018;49(3):660–6. 29459390 10.1161/STROKEAHA.117.020315

[60] Jang KM, Choi HH, Nam TK, Byun JS. Clinical outcomes of first-pass effect after mechanical thrombectomy for acute ischemic stroke: A systematic review and meta-analysis. Clin Neurol Neurosurg. 2021;211:107030. 34823155 10.1016/j.clineuro.2021.107030

[61] Boodt N, van Snouckaert PRW, Hund HM, Fereidoonnezhad B, McGarry JP, Akyildiz AC, et al. Mechanical characterization of thrombi retrieved with endovascular thrombectomy in patients with acute ischemic stroke. Stroke. 2021;52(8):2510–7. 34078112 10.1161/STROKEAHA.120.033527 PMC8312567

[62] Maekawa K, Shibata M, Nakajima H, Mizutani A, Kitano Y, Seguchi M, et al. Erythrocyte-Rich thrombus is associated with reduced number of maneuvers and procedure time in patients with acute ischemic stroke undergoing mechanical thrombectomy. Cerebrovasc Dis Extra. 2018;8(1):39–49. 29402828 10.1159/000486042 PMC5836222

[63] Karimian-Jazi K, Vollherbst DF, Schwarz D, Fischer M, Schregel K, Bauer G, et al. MR microscopy to assess clot composition following mechanical thrombectomy predicts recanalization and clinical outcome. J Neurointerv Surg. 2024;16(8):830–7. 37527928 10.1136/jnis-2023-020594

[64] Nogueira RG, Pinheiro A, Brinjikji W, Abbasi M, Al-Bayati AR, Mohammaden MH, et al. Clot composition and recanalization outcomes in mechanical thrombectomy. J Neurointerv Surg. 2024;16(5):466–70. 37419694 10.1136/jnis-2023-020117

[65] Yun J, Kim KH, Kim S, Chung KS, Nam HS, Heo JH, et al. Endovascular thrombectomy in Cancer-Related stroke: comparison of thrombectomy methods. S:VIN. 2025;5(6). 10.1161/SVIN.125.00192. 10.1161/SVIN.125.001926 PMC12697640 41608714

[66] Ozaki T, Nicholson P, Schaafsma JD, Agid R, Krings T, Pikula A, et al. Endovascular therapy of acute ischemic stroke in patients with Large-Vessel occlusion associated with active malignancy. J Stroke Cerebrovasc Dis. 2021;30(2):105455. 33242784 10.1016/j.jstrokecerebrovasdis.2020.105455

[67] Nissen NI, Karsdal M, Willumsen N. Collagens and cancer associated fibroblasts in the reactive stroma and its relation to cancer biology. J Exp Clin Cancer Res. 2019;38(1):115. 30841909 10.1186/s13046-019-1110-6 PMC6404286

[68] Jeon Y, Baik SH, Jung C, Kim JY, Kim BJ, Kang J, Bae HJ, Kim JH. Mechanical thrombectomy in patients with acute cancer-related stroke: Is the stent retriever alone effective? J Neurointerv Surg. 2021;13:318-323. 10.1136/neurintsurg-2020-016144 32586910

